# Genomic predictions to leverage phenotypic data across genebanks

**DOI:** 10.3389/fpls.2023.1227656

**Published:** 2023-08-28

**Authors:** Samira El Hanafi, Yong Jiang, Zakaria Kehel, Albert W. Schulthess, Yusheng Zhao, Martin Mascher, Max Haupt, Axel Himmelbach, Nils Stein, Ahmed Amri, Jochen C. Reif

**Affiliations:** ^1^ Leibniz Institute of Plant Genetics and Crop Plant Research (IPK), Gatersleben, Germany; ^2^ International Center for Agricultural Research in Dry Areas (ICARDA), Rabat, Morocco; ^3^ Center for Integrated Breeding Research (CiBreed), Georg-August-University, Göttingen, Germany

**Keywords:** barley, genebank, genomic prediction, ICARDA, IPK, prediction ability

## Abstract

Genome-wide prediction is a powerful tool in breeding. Initial results suggest that genome-wide approaches are also promising for enhancing the use of the genebank material: predicting the performance of plant genetic resources can unlock their hidden potential and fill the information gap in genebanks across the world and, hence, underpin prebreeding programs. As a proof of concept, we evaluated the power of across-genebank prediction for extensive germplasm collections relying on historical data on flowering/heading date, plant height, and thousand kernel weight of 9,344 barley (*Hordeum vulgare* L.) plant genetic resources from the German Federal Ex situ Genebank for Agricultural and Horticultural Crops (IPK) and of 1,089 accessions from the International Center for Agriculture Research in the Dry Areas (ICARDA) genebank. Based on prediction abilities for each trait, three scenarios for predictive characterization were compared: 1) a benchmark scenario, where test and training sets only contain ICARDA accessions, 2) across-genebank predictions using IPK as training and ICARDA as test set, and 3) integrated genebank predictions that include IPK with 30% of ICARDA accessions as a training set to predict the rest of ICARDA accessions. Within the population of ICARDA accessions, prediction abilities were low to moderate, which was presumably caused by a limited number of accessions used to train the model. Interestingly, ICARDA prediction abilities were boosted up to ninefold by using training sets composed of IPK plus 30% of ICARDA accessions. Pervasive genotype × environment interactions (GEIs) can become a potential obstacle to train robust genome-wide prediction models across genebanks. This suggests that the potential adverse effect of GEI on prediction ability was counterbalanced by the augmented training set with certain connectivity to the test set. Therefore, across-genebank predictions hold the promise to improve the curation of the world’s genebank collections and contribute significantly to the long-term development of traditional genebanks toward biodigital resource centers.

## Introduction

Collections of plant genetic resources (PGRs) are a valuable source of diversity that provides the basis for developing disease-resistant, nutrient-dense, and climate-resilient crop varieties (Hoisington et al., 1999). However, given the vastness of genebank holdings, selecting the most suitable accessions with specific desirable traits for breeding is challenging. The limited passport information and basic phenotypic characterization for important agronomic traits and the lack of robust and cost-efficient phenotyping capacities are currently chief among the bottlenecks restricting the full exploitation of plant genetic resources (Furbank and Tester, 2011; McCouch et al., 2013; Anglin et al., 2018). Because the characterization of entire collections in genebanks is resource-intensive and time-consuming, high-throughput genomic tools have been proposed to leverage the potential of genebank collections. Tremendous advances in genotyping technology sharply reduced the cost of genotyping, facilitating the generation of large-scale sequencing and genotyping datasets for entire genebank collections (Kilian and Graner, 2012). Pioneering international projects have thus genomically characterized comprehensive collections of genetic resources and are making this information available in biodigital resource centers. For instance, the Federal Ex situ Genebank hosted at the Leibniz Institute of Plant Genetics and Crop Plant Research (IPK) in Gatersleben, Germany, has genotyped its barley collection (22,626 accessions) using a genotyping by sequencing (GBS) platform (Milner et al., 2019). This information and the plant material can be accessed and visualized through the BRIDGE web portal (https://bridge.ipk-gatersleben.de/). The Seeds of Discovery initiative, which aims to promote the effective use of PGR, has characterized 37% and 66% of International Maize and Wheat Improvement Center (CIMMYT) and International Center for Agriculture Research in the Dry Areas (ICARDA) wheat genebank accessions, respectively, using sequencing-based diversity array technology (DArTseq; Sansaloni et al., 2020).

Systematic valorization of the produced genomic data has made rapid advances in subsequent genomic studies and breeding purposes. In combination with the genomic profile, genebank’s comprehensive historical phenotypic data, accumulated over the years, provided useful information about genetic gaps in collections (Volk et al., 2021). Different genomic approaches were widely implemented to close the gap between genebank management and prebreeding. For instance, genome-wide prediction has been proposed to effectively impute phenotypic information for entire genebank collections based on representative subsamples of entire collections for which genotypes and phenotypes have been recorded. These panels can be used as training populations for genotyped accessions lacking phenotypic records (Yu et al., 2016). Several studies have demonstrated the potential of using genome-wide prediction for genebank collections (Crossa et al., 2016; Kehel et al., 2020; Jiang et al., 2021; Schulthess et al., 2022). Alternatively, genome-wide prediction based on training datasets generated in other genebanks has the potential to increase the attractiveness of collections around the world by providing information to users for a wide range of traits. This approach has been used in a study to predict yield breeding values for winter wheat accessions maintained at INRAE (L’institut national de recherche pour l’agriculture, l’alimentation et l’environnement) using IPK-PGRs as training data, but the validation of the predictions was not implemented (Schulthess et al., 2022).

To fill this gap, we integrated our study data across the two important barley collections maintained at ICARDA and IPK. Prior to applying genomic prediction, a strategic pipeline to curate the non-orthogonal historical data was implemented for the IPK collection (González et al., 2018b). The same rigorous quality assessment including plausibility checks, outlier corrections, and bias estimation due to the historical seed regeneration patterns was applied independently for each of the winter, spring, and facultative ICARDA barley populations. Therefore, our study makes use of comprehensive historical phenotypic and genomic data of 9,344 and 1,089 barley accessions from IPK and ICARDA genebanks, respectively. The main goal was to evaluate the potential and limitations of genome-wide predictions across genebanks using IPK and ICARDA historical phenotypic data. In particular, our objectives were to 1) assess the quality of ICARDA historical data for heading date (HD), plant height (PH), and thousand kernel weight (TKW); 2) study the population structure of both IPK and ICARDA collections; 3) examine the prediction ability of the same given traits within ICARDA population defined by growth habit and row type; 4) assess the benefits of across-genebank prediction in imputing phenotypes of ICARDA accessions relying only on the IPK genebank (one-sided approach) or 5) on a combined IPK-plus-ICARDA training set (integrated approach).

## Materials and methods

### Phenotypic data records

Field experiments for 16,554 ICARDA barley accessions were performed from 1983 to 2012 in Tel Hadya, Syria (latitude 36.01°40′N, longitude 36.56°20′E, 284 m.a.s.l) and from 2016 to 2019 in Merchouch, Morocco (latitude 33°36′N, longitude 6°43′W, 394 m.a.s.l.) (**Supplementary Table 1**). Across traits, 48,882 data points were recorded for HD, PH, and TKW, mostly in unreplicated field trials. Heading date was recorded as the number of days when 50% of the plants in each observation plot have emerged to 75% from the flag leaf sheath (Z57 stage according to Zadoks et al., 1974) starting from the date of sowing. PH was measured from the ground level to the top of the spike, including awns, at the end of the flowering period. TKW was determined in grams by weighing a representative sample of grains harvested at ∼12.5% moisture basis, counting grains, and extrapolating the weight to 1,000 grains. Approximately 50% of the accessions were phenotyped for HD and PH for 2 years, while TKW had the lowest percentage (18.4%) of accessions with 2 years of observations. ICARDA collection included 7,576 spring, 881 winter, and 4,164 facultative barley accessions. In addition, approximately 14% of the accessions (2,369) have ambiguous growth habit records, and 9% (1,566) have no available information. The majority (72%) of the accessions were six-rowed type, while two-rowed barley represented 24% of the total collection. The classification of winter, spring, and facultative ICARDA accessions was not derived from a premeditated experimental design. Rather, the barley accessions were sown toward the end of November or the beginning of December, without deliberate consideration of vernalization treatments to induce the winter type. The accessions’ responses to prevailing environmental conditions were closely monitored and meticulously recorded. Under favorable circumstances, the presence of winter types became evident, as all spring accessions demonstrated successful progression to the heading and maturity stages. Nevertheless, in several instances, the natural ambient temperatures failed to provide sufficient vernalization, leading to the classification of certain accessions as winter or facultative types due to their inability to reach the heading stage, with only a limited number of plants in the plots achieving successful maturity. However, an interesting aspect emerged concerning facultative accessions being able to integrate cues from both winter and spring conditions, resulting in a marginally prolonged period to reach the heading stage. This growth type exhibits cold tolerance and can set seeds without the need for vernalization, indicating their adaptability to varying environmental cues.

Phenotypic data from IPK included information on 6,957 spring and 2,387 winter barley accessions collected from the IPK campus (Gatersleben, Germany; latitude 51°49′22.5″N, longitude 11°16′40.6″E, 110.5 m.a.s.l.). Spring barley subpopulation included 4,425 six-rowed and 2,532 two-rowed accessions. Winter subpopulations included 1,901 and 486 accessions of six-rowed and two-rowed accessions, respectively (**Supplementary Table 2**). The accessions were phenotyped for flowering time (FT), PH, and TKW (referred also to as thousand grain weight) during their regeneration in the past seven decades, and the associated phenotypic information was previously described in detail by González et al. (González et al., 2018a; González et al., 2018b). FT was recorded as the number of days when 50% of the plants in each observation plot reached the flowering counting from January 1 of each year for winter types and from the sowing date onward in the case of spring types. The flowering stage for both winter and spring corresponds to stage Z65 (Zadoks et al., 1974). High correlations have been reported between flowering time and heading date as a result of their closeness during crop phenology (Gol et al., 2021; Celestina et al., 2023). Therefore, we considered FT from IPK accessions as a proxy trait for HD of ICARDA material in across-genebank prediction. PH and TKW of IPK accessions were assessed as previously described for the ICARDA genebank. Each of the three traits was analyzed using a linear mixed model for quality assessment routines and performance estimation (González et al., 2018a; González et al., 2018b). Outlier removal led to high heritability estimates exceeding 0.8, and the resulting best linear unbiased estimations (BLUEs) for each of the traits were used in this study.

### Phenotypic data quality assessment and performance estimates for ICARDA material

Phenotypic data analyses for the ICARDA collection were performed following the methods specified for the IPK genebank (González et al., 2018a; González et al., 2018b). Analyses were conducted for winter, spring, and facultative accessions separately, and the accessions with non-unique records of growth class were excluded. The following linear mixed model was applied:


$$y=1_{m}\mu +Z_{1}g+Z_{2}t+Z_{3}i+e,\;\left(1\right)$$

where y is the *m*-dimensional vector of phenotypic records, μ is the common intercept term, g is the *n*-dimensional vector of genotypic effects, $Z_{1}$ is an m×n design matrix allocating each record to the corresponding accession, t is the *l*-dimensional vector of year effects, $Z_{2}$ is an m×l design matrix allocating each record to the corresponding year, i is the *s*-dimensional vector of genotype-by-year interaction effects, $Z_{3}$ is the corresponding m×s design matrix, and e is the residual term. In Equation 1, we assumed that μ is a fixed parameter, while the remaining components are random in the way $g∼N\left(0,I\sigma_{g}^{2}\right)$ , $t∼N\left(0,I\sigma_{t}^{2}\right)$ , $i∼N\left(0,I\sigma_{i}^{2}\right)$ , and $e∼N\left(0,I\sigma_{e}^{2}\right)$ . The broad-sense heritability was estimated as $h^{2}=\frac{{\hat{\sigma }}_{g}^{2}}{{\hat{\sigma }}_{g}^{2}+\frac{{\hat{\sigma }}_{i}^{2}}{q}+\frac{{\hat{\sigma }}_{e}^{2}}{p}}$ , where ${\hat{\sigma }}_{g}^{2}$ , ${\hat{\sigma }}_{t}^{2}$ , ${\hat{\sigma }}_{i}^{2}$ , and ${\hat{\sigma }}_{e}^{2}$ are the estimates of the corresponding variance components, q is the harmonic mean of the number of evaluated years per genotype, and p is the harmonic mean of the number of replicates per genotype (Holland et al., 2003).

Model 1 was also used for the outlier test with slightly different assumptions, that is, treating g as a vector of fixed effects instead of random. The residuals were first standardized by the rescaled median absolute deviation from the median, and then a Bonferroni–Holm test was applied to flag the outliers (Bernal-Vasquez et al., 2016). A data point was declared as an outlier by the implemented test according to a predefined significance threshold of p-value < 0.05. After removing the outliers from the initial dataset, model 1 was fitted again to recompute variance components and broad-sense heritabilities as well as to calculate the genotypic BLUEs. For BLUE computation, the same assumptions in model 1 as specified for outlier correction were considered. All mixed models for phenotypic analyses were fitted using the ASReml R package version 4 (Butler et al., 2017).

### Genomic data

A total of 22,626 accessions from the IPK were previously fingerprinted using GBS technology (Milner et al., 2019). In this study, 1,803 ICARDA accessions were characterized based on the same method: briefly, DNA was digested with *Pst*I and *Msp*I (New England Biolabs) restriction enzymes, and sequencing was performed with Illumina HiSeq 2500. Read mapping and variant calling were performed essentially as described by Milner et al. (2019). After adapter trimming with cutadapt (Martin, 2011), reads were aligned to the MorexV3 reference genome sequence assembly with BWA-MEM (Li, 2013). Alignment records were converted to Binary Alignment/Map format with samtools and sorted with Novosort (http://www.novocraft.com/products/novosort/). Variant calling was performed with bcftools (Li, 2011). Variant matrices were filtered and formatted with a custom script (ipk_dg_public, 2018) prior to input into R via the SNPRelate package (Zheng et al., 2012). Only bi-allelic single-nucleotide polymorphisms (SNPs) with less than 10% heterozygous calls were retained. After this filtering, GBS profiles were integrated with the BLUEs (after outlier correction) of 9,344 IPK and 1,116 ICARDA accessions with known row-type information (**Supplementary Tables 1, 2**). In this integrated dataset, a final total of 27,610 SNPs was retained after applying quality control criteria (call rate >0.95 and minor allele frequency (MAF) >0.05).

### Population structure and genome-wide predictions

Genetic relationships among 1,116 ICARDA and 9,344 IPK accessions were investigated using a principal coordinate analysis (PCoA) based on the Euclidean distances computed from markers. PCoA was performed using the “ecodist” R package (version 2.0.9).

For genomic predictions, 27 ICARDA accessions having phenotypic information but belonging to the 2RF row-type group were excluded because this row type did not exist among IPK accessions. Row-type 2RF comprises barley accessions in which each spikelet contains two rows of seeds, the two outer rows of seeds being larger and more prominent, forming ridges along the length of the spikelet. The inner seeds may be smaller or less developed. The distinction between the two-rowed and 2RF classifications is based on the level of detail provided about the seed arrangement within the spikelet on the barley head. Among the genotyped ICARDA accessions, 1,071, 1,057, and 1,081 accessions were phenotyped for HD, PH, and TKW, respectively. For IPK, 9,341, 9,298, and 7,575 genotyped accessions had BLUEs for FT, PH, and TKW, respectively. Three different genome-wide prediction models were applied: 1) genomic best linear unbiased prediction (GBLUP; VanRaden, 2008), 2) extended genomic best linear unbiased prediction (EGBLUP; Jiang and Reif, 2015), and 3) reproducing kernel Hilbert space regression (RKHS; Gianola and van Kaam, 2008).

The GBLUP model exploits the additive effects of all markers and has the following form:


(2) $$y=X\beta +g_{A}+e$$

where y is the *n*-dimensional vector of BLUEs obtained from the phenotypic data analyses, β is the *k*-dimensional vector of fixed effects including covariates (if any) and the intercept, X is the corresponding design matrix (if there are no covariates in the model, then β=μ the common intercept and $X=1_{n}$ a column vector of ones), $g_{A}∼N\left(0,G_{A}\sigma_{g}^{2}\right)$ is the *n*-dimensional random vector of (additive) genetic values, and $e∼N\left(0,I\sigma_{e}^{2}\right)$ is the residual. $G_{A}=ZZ^{"}$ is the VanRaden G-matrix, where $Z=M/\sqrt{c}$ , M is the n×s matrix of marker profiles coded as 2−2p , 1−2p , and −2p ( p is the allele frequency), $c=\sum_{i=1}^{s}2p_{i}\left(1-p_{i}\right)$ , and s is the number of markers.

The EGBLUP model is an extension of the GBLUP model by considering additive-by-additive epistatic effects between all pairs of markers:


(3) $$y=X\beta +g_{A}+g_{AA}+e$$

where $g_{AA}∼N\left(0,G_{AA}\sigma_{g}^{2}\right)$ is the *n*-dimensional random vector of additive-by-additive genetic values, while all other notations are the same as in the GBLUP model. The epistatic covariance matrix was calculated as follows (Jiang and Reif, 2020):


(4) $$G_{AA}=\frac{1}{2}\left(G_{A}\circ G_{A}-\left(Z\circ Z\right){\left(Z\circ Z\right)}^{"}\right)$$

where ∘ denotes the Hadamard product of matrices.

The RKHS model originated from a semi-parametric approach, but its form is similar to the GBLUP model with a different covariance matrix (de Los Campos et al., 2010). The RKHS model exploits additive and epistatic effects among markers up to any order, but the weights for different orders of epistasis were implicitly fixed (Jiang and Reif, 2015). In our implementation, we followed the “kernel averaging” approach (de Los Campos et al., 2010); i.e., we considered the following:


(5) $$y=X\beta +g_{1}+g_{2}+g_{3}+e$$

where $g_{i}∼N\left(0,K_{i}\sigma_{g_{i}}^{2}\right)$ and other notations are the same as specified in GBLUP. The element in the j th row and k th column of $K_{i}$ is calculated as $\text{exp }\left(-h_{i}\frac{\sum_{l=1}^{m}{\left(x_{jl}-x_{kl}\right)}^{2}}{m}\right)$ , where $x_{jl}$ is the l th marker profile of the j th individual, and $\left(h_{1},h_{2},h_{3}\right)=\left(0.1,0.5,1\right)$ .

All genomic prediction models were implemented using the R package BGLR (Pérez and de Los Campos, 2014).

### Establishing genebank genomic prediction scenarios for ICARDA accessions

We evaluated the prediction ability of the GBLUP, EGBLUP, and RKHS models using the following scenarios for ICARDA accessions.

#### Within-genebank prediction

Fivefold cross-validation was applied separately within each growth class among ICARDA accessions. For each growth class, accessions were randomly divided into five subsets, each with balanced proportions of accessions sampled from both row types, of which four subsets served as the training set with the remaining as the test set. The sampling was repeated 20 times.

#### One-sided across-genebank prediction

Here, only information from the IPK barley collection was used to predict ICARDA accessions. Five sub-scenarios, 2a–e, with different combinations of training and test sets were considered to assess prediction ability in the case of merging the different row types and within each row-type subpopulation (**Supplementary Table 3**):

Scenario 2a: Using winter IPK accessions as the training set to predict winter ICARDA accessions.
Scenario 2b: Using spring IPK accessions as the training set to predict spring ICARDA accessions.

Since the number of IPK facultative types was small as compared with the rest of the two other growth classes (spring and winter), we decided not to treat them as a separate group to predict the facultative ICARDA accessions, but instead, we opted for the following scenarios:

Scenario 2c: Using winter IPK accessions to predict ICARDA facultative accessions.
Scenario 2d: Using spring IPK accessions to predict ICARDA facultative accessions.
Scenario 2e: Pooling together winter and spring IPK accessions to predict ICARDA facultative accessions.

#### Integrated across-genebank prediction

In this approach, the same sub-scenarios described in scenario 2 were implemented with a slight adjustment of the training sets. The phenotypic records of 30% of ICARDA accessions were integrated with the respective phenotypic records of the IPK dataset to predict the rest of ICARDA accessions. Twenty random samplings were considered and performed separately.

For each scenario, each of the three genomic prediction models was implemented twice, one ignoring the row-type subgroup information and the other modeling the row type as a fixed covariate. The influence of the row type on the prediction ability was investigated by comparing the prediction abilities that resulted from these two cases. For all three scenarios, the prediction ability was estimated as the correlation between the observed and predicted values of all accessions in the test set. In addition, the prediction ability for each row-type subgroup was also calculated separately. The standard error was estimated using a bootstrap approach with 1,000 samplings.

## Results

### Broad genetic variation observed for the assessed traits

Linear mixed models combined with rigorous quality assessment were implemented for historical IPK data and described in detail in recent works (González et al., 2018a; González et al., 2018b). Briefly, based on a two-step quality control that considers plausibility checks of trait values and outlier corrections, high heritability estimates (above 0.8) were obtained for the traits under consideration (**Supplementary Table 4**). Moreover, heritability increased by 17% by removing a maximum of 2.5% outliers for the IPK collection. The same strategy was applied in the analysis of historical phenotypic data from ICARDA. The outlier correction resulted in the exclusion of up to 1.74% of the total accessions and increased the heritability by up to 41%, depending on the trait and growth class (**Table 1**). Heritabilities observed for the ICARDA barley accessions were lower than those for IPK (**Table 1**; **Supplementary Table 4**). This discrepancy in heritabilities can be attributed to several factors, including disparities in phenotyping conditions and prevailing environmental stresses. It is noteworthy that ICARDA accessions were grown in a harsh environment characterized by frequent drought and heat stress. Such challenging conditions can significantly impact the phenotypic expression of traits, resulting in lower heritability estimates. The stress-induced variability can mask the genetic component, leading to decreased heritability values. In contrast, the German environment provided an optimum growing condition for the IPK accessions and, hence, led to higher heritabilities by reducing the environmental noise, which could otherwise affect the genetic expression of the traits. TKW was the most heritable trait in each growth class with heritability estimates exceeding 0.65 (**Table 1**). In contrast, HD and PH had moderate heritabilities with values ranging from 0.32 to 0.50. Of the total number of accessions (two- and six-rowed row types) with both phenotypic and genotypic data (1,264), 1,089 accessions were retained after outlier correction (**Supplementary Table 5**). These accessions were used for genomic prediction analyses.

**Table 1:** The number of outliers for each trait in each growth class and the influence of removing outliers on the estimated broad-sense heritability for ICARDA collection.

Trait	Growth class	$N_{data}$	$N_{outlier}$	$N_{geno}$	$N_{geno\_out}$	${\hat{h}}^{2}$	${\hat{h}}_{out}^{2}$	$\text{Δ}{\hat{h}}^{2}$ (%)
**HD**	Winter	3,736	45 (1.20%)	2,366	18 (0.76%)	0.38	0.42	11.0
	Facultative	6,742	144 (2.14%)	4,158	51 (1.23%)	0.23	0.32	41.0
	Spring	12,407	354 (2.85%)	7,572	112 (1.48%)	0.35	0.46	31.6
**PH**	Winter	3,727	86 (2.31%)	2,360	41 (1.74%)	0.40	0.50	23.6
	Facultative	6,730	107 (1.59%)	4,158	35 (0.84%)	0.29	0.35	19.5
	Spring	12,324	271 (2.20%)	7,563	84 (1.11%)	0.36	0.46	26.0
**TKW**	Winter	3,095	11 (0.36%)	2,252	5 (0.22%)	0.70	0.75	8.0
	Facultative	4,655	19 (0.41%)	4,032	9 (0.22%)	0.55	0.72	31.4
	Spring	8,794	25 (0.28%)	7,538	3 (0.04%)	0.59	0.65	10.6

$N_{data}$ , the number of phenotypic records; $N_{outlier}$ , the number of outliers; $N_{geno}$ , the number of accessions; $N_{geno\_out}$ , the number of accessions that were identified as outliers; ${\hat{h}}^{2}$ , the estimated heritability; ${\hat{h}}_{out}^{2}$ , the re-estimated heritability after removing all outliers; $\Delta {\hat{h}}^{2}$ , the difference between the estimated heritability after and before removing all outliers (in percentage); HD, heading date (days); PH, plant height (cm); TKW, thousand kernel weight (g).

**Figure 1:**
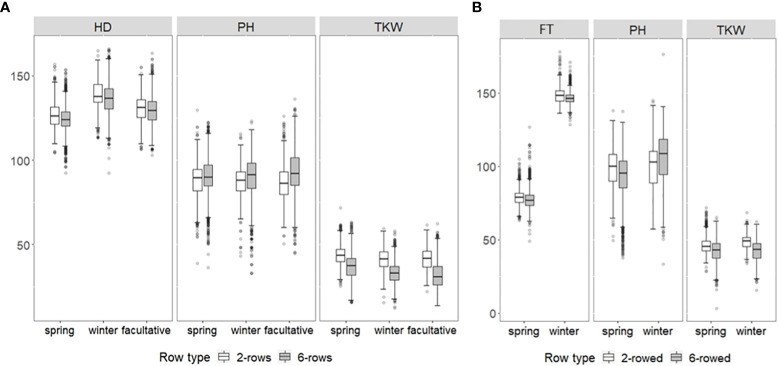
Box–whisker plots showing the distribution of best linear unbiased estimations of heading date (HD; days), flowering time (FT; days), plant height (PH; cm), and thousand kernel weight (TKW; g) of up to **(A)** 1,089 ICARDA and **(B)** 9,344 IPK accessions for two-rowed (white boxes) and six-rowed subgroups (gray boxes). Distribution is shown separately for winter, spring, and facultative barley.

Regarding BLUEs, wide phenotypic variation was observed in each growth class for both genebank collections (**Figure 1**). For ICARDA accessions in general, HD was between 92.27 days and 165.92 days, PH between 33.19 cm and 136.05 cm, and TKW between 12.43 g and 71.7 g (**Figure 1A**). For IPK accessions, FT varied between 128.31 days and 178.15 days, PH between 50.44 cm and 176.51 cm, and TKW between 15.64 g and 68.44 g (**Figure 1B**). Except for FT across growth habits of IPK accessions, no significant average differences (p-value ≥ 0.05) were observed between either growth habits or row types within IPK and ICARDA genebanks indicating that the genotyped fractions of both collections cover a similar space of phenotypic diversity, at least for the assessed historic traits. However, the significantly earlier average in FT of IPK spring compared to winter accessions (Δ = 69.9 days, p-value< 2.2e−16) is most likely due to the different reference day, i.e., January 1 (winter types) *vs.* sowing date (spring types), used to express this trait. Moreover, no significant average differences were observed between IPK and ICARDA genebanks regarding PH and TKW (p-value ≥ 0.05).

### The global diversity of the ICARDA collection was fully covered by the IPK collection

**Figure 2:**
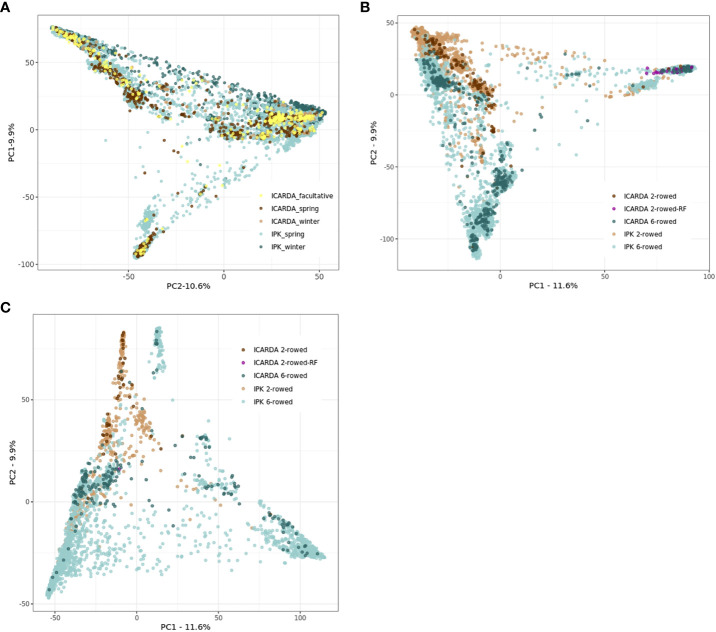
Principal coordinate analyses (PCoAs) based on the Euclidean distances were estimated using 27,610 single-nucleotide polymorphisms (SNPs), 1,116 ICARDA, and 9,344 IPK accessions **(A)**. Spring **(B)** and winter **(C)** populations were plotted separately for ICARDA and IPK accessions. **(A)** Principal coordinate analyses (PCoAs) for both ICARDA and IPK accessions. **(B)** Principal coordinate analyses (PCoAs) for spring ICARDA and IPK accessions. **(C)** Principal coordinate analyses (PCoAs) for winter ICARDA and IPK accessions.

The genetic structure of ICARDA and IPK collections was investigated through a PCoA based on the Euclidean distance matrix estimated using 27,610 SNPs. At first glance, the accessions derived from the ICARDA genebank seem to occupy a relatively small area of IPK diversity space. However, this result should be interpreted carefully since only 1,116 genotyped ICARDA accessions were used in this analysis (**Figure 2A**). Extensive genotyping of the ICARDA collection is necessary to highlight the specifics of that collection. The facultative accessions of the ICARDA collection did not form a clearly delineated group from the rest of the accessions (**Figure 2A**). In the spring and winter barley subpopulations, the six-rowed and two-rowed accessions were clearly separated with some exceptions pointing to admixture (**Figures 2B, C**).

### Within-genebank genomic prediction of ICARDA accessions

**Figure 3:**
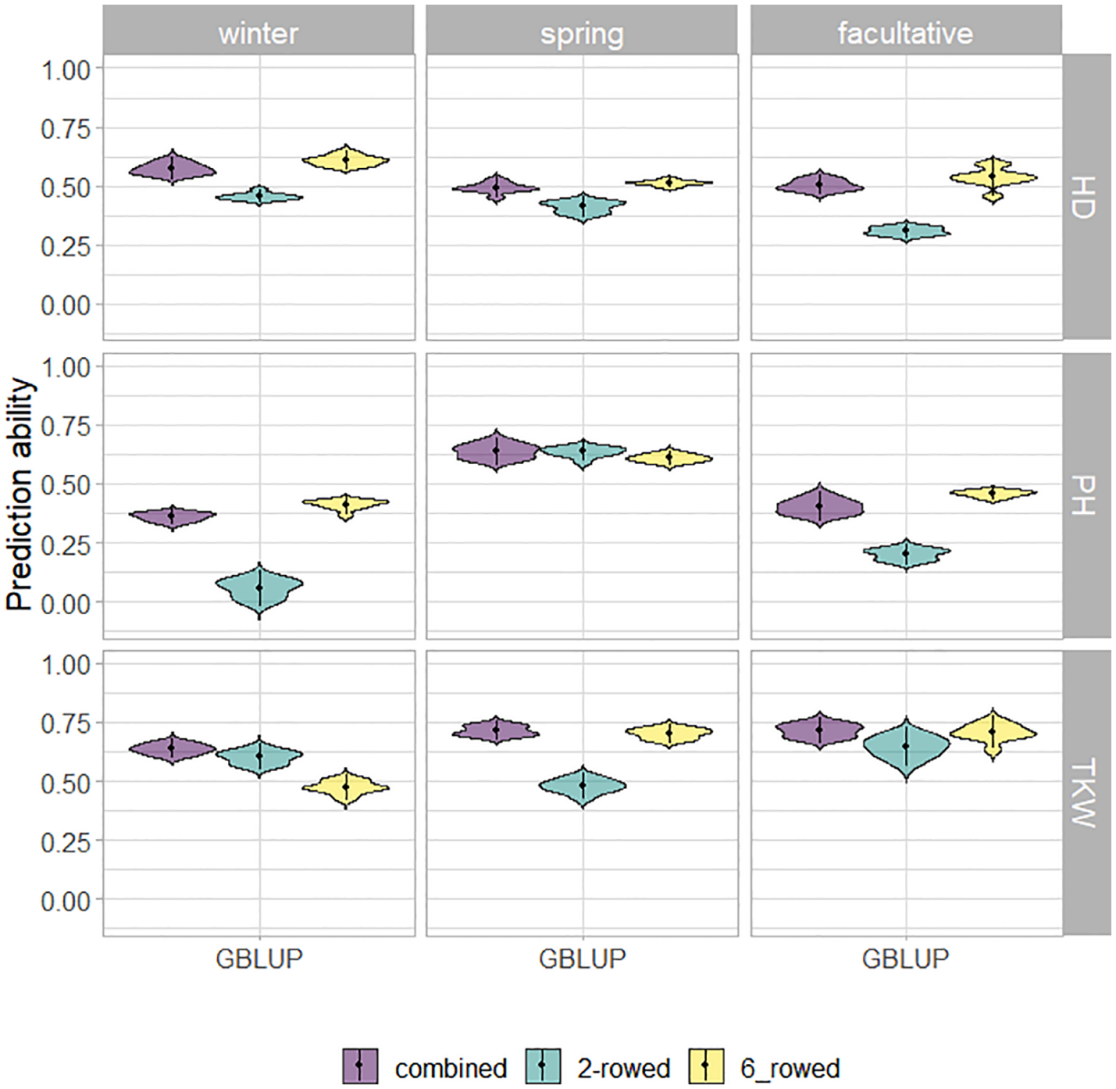
Fivefold cross-validated prediction abilities of genomic best linear unbiased prediction (GBLUP) for heading date (HD; days), plant height (PH; cm), and thousand kernel weight (TKW; g) obtained within ICARDA genebank modeling the row type as covariate (RT).

**Figure 4:**
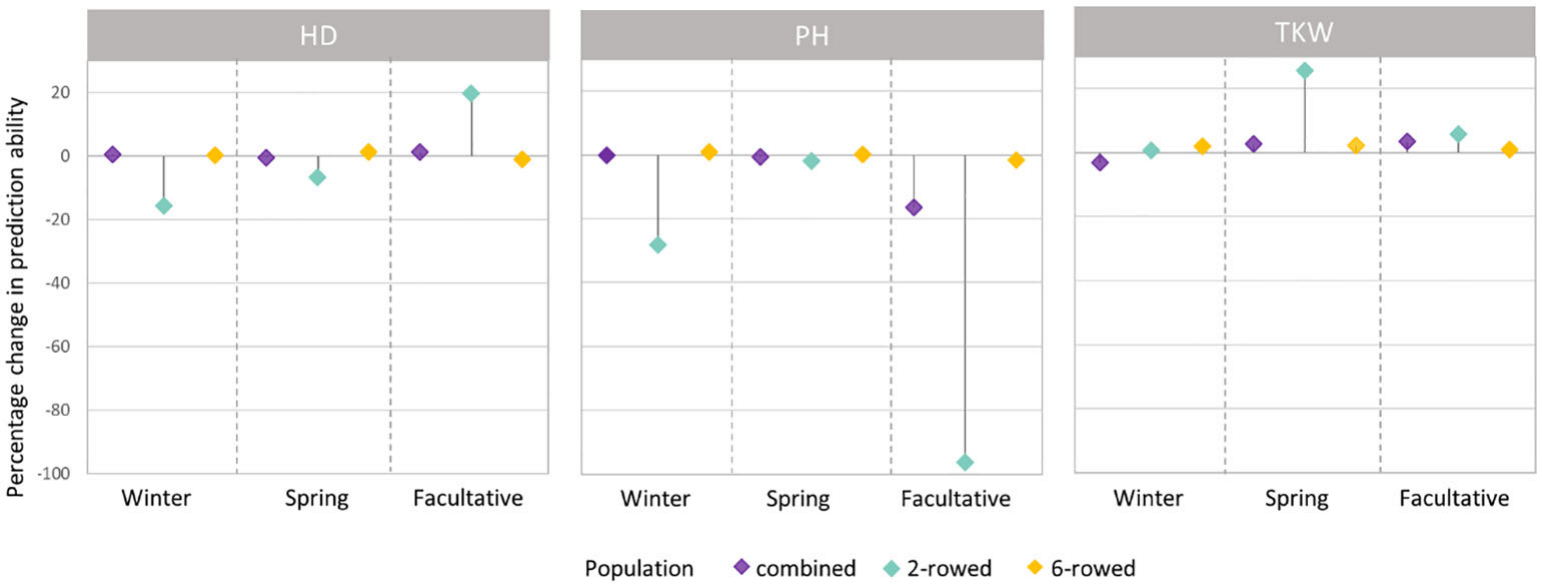
Percentage change (%) in average prediction abilities from modeling the row type as a covariate within the population of ICARDA accessions using genomic best linear unbiased prediction (GBLUP) over omitting the row-type covariate for heading date (HD; days), plant height (PH; cm), and thousand kernel weight (TKW; g) according to different growth habits. Positive (negative) changes correspond to improvements (declines) in prediction ability.

The fivefold cross-validated prediction abilities within the population of ICARDA accessions varied widely among traits and populations defined by growth habits and row types (**Figure 3**). According to regression analysis, the heritability (**Table 1**) and size (**Supplementary Table 5**) of populations defined by growth habits explained 47% and 41% of the variation in the average prediction abilities, respectively. Overall, the prediction abilities depended only slightly on the choice of the prediction model: on average, GBLUP outperformed EGBLUP and RKHS by 2.79% and 5.39%, respectively (**Supplementary Table 6**). Therefore, GBLUP was chosen as the base model for the benchmark scenario. When row types were merged in a combined training set, average GBLUP prediction abilities improved 32.64% for two-rowed types, but only 0.27% for six-rowed accessions (**Figure 3**). The six-rowed populations were 3.9-fold larger than the two-rowed subpopulations, which can explain why the two-rowed benefited more from a combined training set (**Supplementary Table 5**). In most cases, modeling the row type as a covariate in genome-wide predictions either did not change or even decreased average prediction abilities, with TKW and HD predictions for two-rowed accessions being the most notable exceptions (**Figure 4**). From these optimization results, it was decided to omit the row-type covariate from all prediction scenarios.

### Accurate prediction of ICARDA accessions relying on training sets exclusively composed of IPK material is trait-dependent

**Figure 5:**
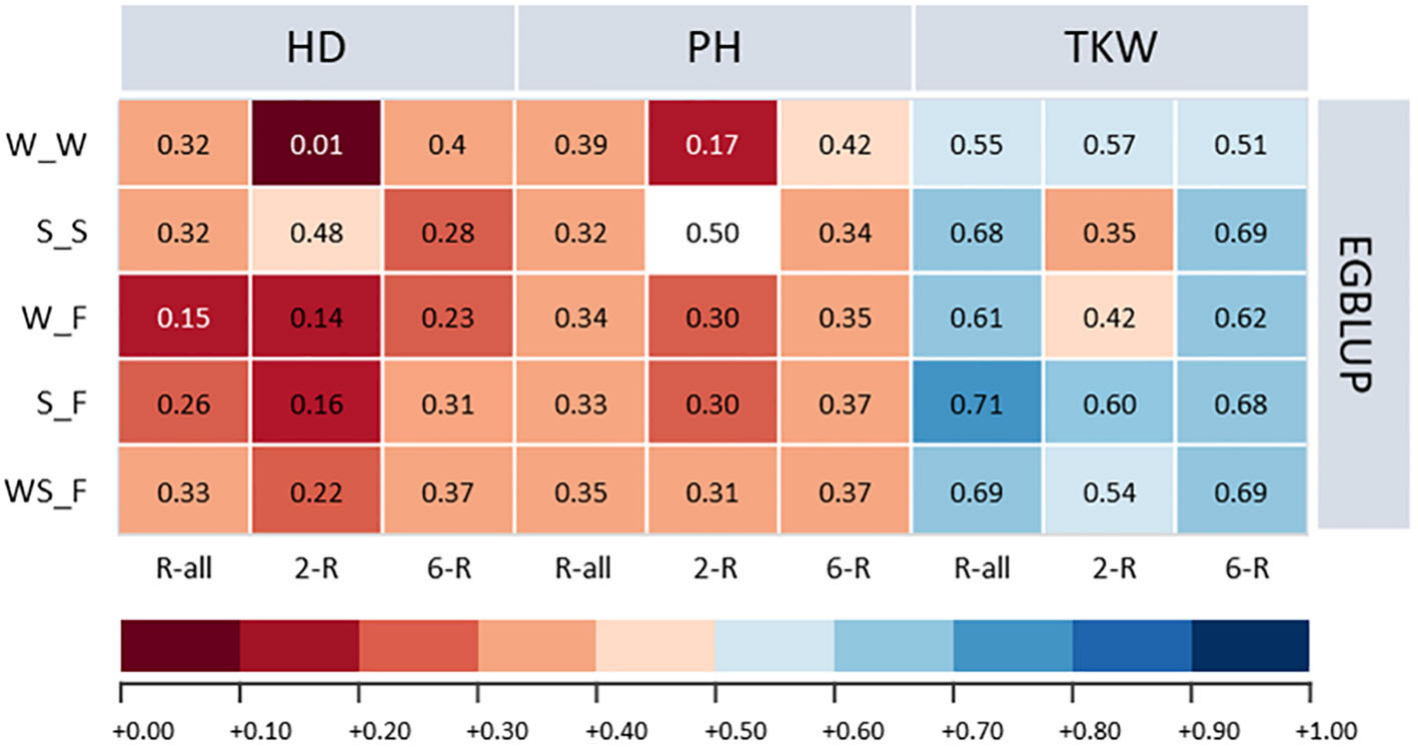
Prediction abilities of extended genomic best linear unbiased prediction (EGBLUP) method for heading date (HD; days), plant height (PH; cm), and thousand kernel weight (TKW; g) of ICARDA accessions obtained by applying a one-sided across-genebank prediction approach. W_W, winter to winter (scenario 2a); S_S, spring to spring (scenario 2b); W_F, winter to facultative (scenario 2c); S_F, spring to facultative (scenario 2d); WS_F, winter and spring to facultative (scenario 2e).

With some marginal trait-specific differences, the average prediction ability for ICARDA accessions of the one-sided across-genebank prediction approach was for EGBLUP 9.67% and 0.77% higher compared with GBLUP and RKHS, respectively (**Supplementary Table 7**). Therefore, we focused the following on the results for EGBLUP (**Figure 5**). Because of the small number of facultative types in the IPK collection, we first studied whether spring, winter, or a combined population is best suited to predict the performance of the facultative ICARDA accessions. Interestingly, a combined spring/winter IPK training population yielded the highest prediction ability of the facultative ICARDA accessions, with minor deviations observed for TKW, as compared to the spring-to-facultative scenario.

Because TKW showed the highest heritability among ICARDA accessions, considerable predictive ability was shown for the given trait with small deviations from the within-genebank prediction scenario (**Supplementary Table 4**; **Supplementary Figure 1**). For PH, the one-sided across-genebank predictions of ICARDA spring and facultative accessions were in most cases lower than within the genebank but vice versa for the winter-to-winter scenario. In contrast, the prediction of HD, with the exception of the two-rowed spring type, was much less accurate across than within the genebank: the difference in prediction ability was rather large ranging from −97.37% to −28.38% (**Supplementary Figure 1**). Consequently, predictions across genebanks using the one-sided approach were not always fully resilient, which may be resolved via prediction using an integrated training population across genebanks.

### Enhanced prediction ability by using an integrated approach across IPK and ICARDA genebanks

**Figure 6:**
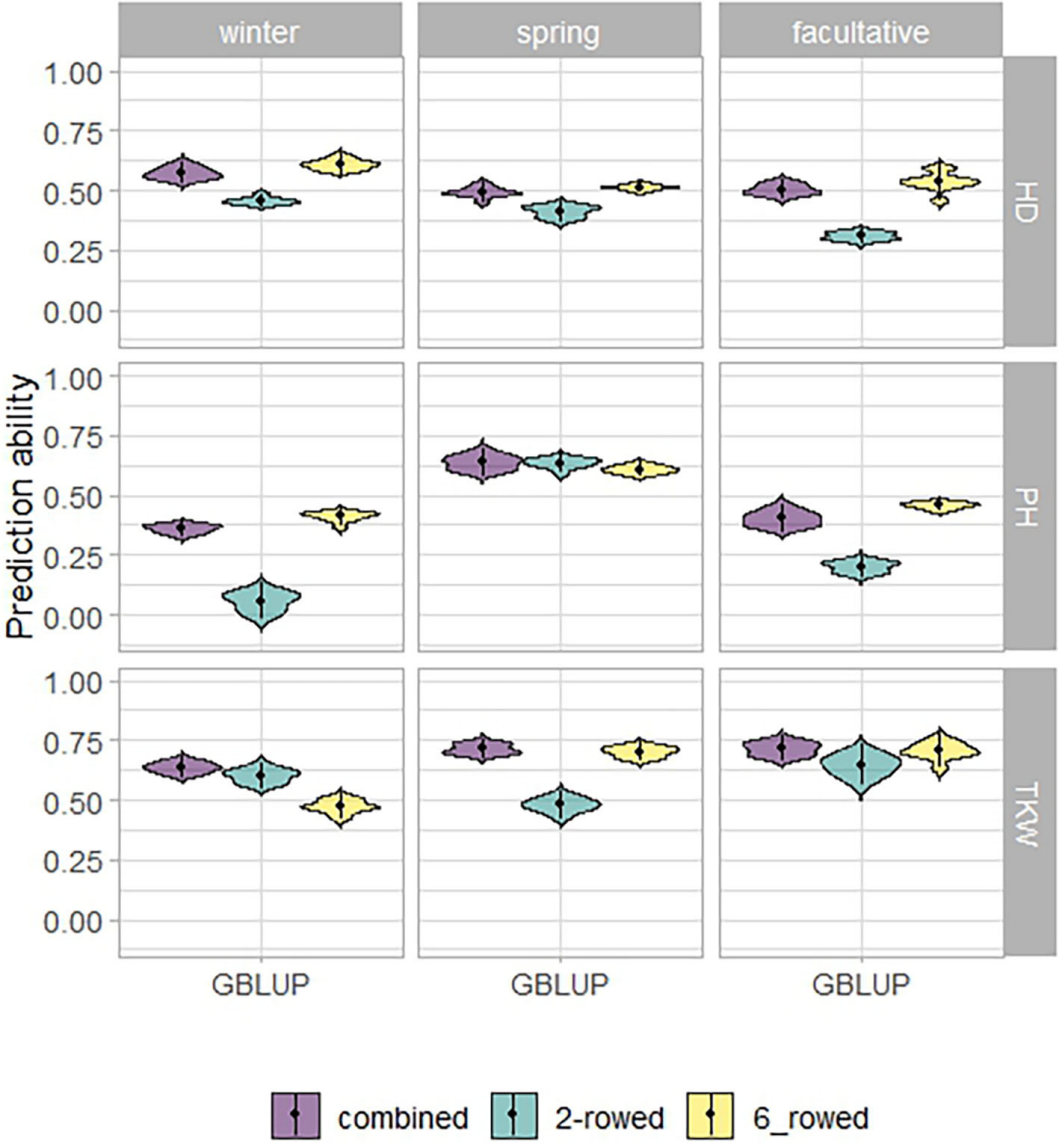
Prediction abilities to reproduce kernel Hilbert space regression (RKHS) for heading date (HD; days), plant height (PH; cm), and thousand kernel weight (TKW; g) of ICARDA accessions in the integrated across-genebank prediction scenario. W_W, winter to winter (scenario 2a); S_S, spring to spring (scenario 2b); WS_F, winter and spring to facultative (scenario 2e).

Using a training population combining IPK and ICARDA accessions to predict the performance of an ICARDA test population revealed that the average prediction abilities based on GBLUP proved to be the least accurate model. RKHS achieved 6.10% higher average prediction abilities than GBLUP and improved by 1.87% compared to EGBLUP (**Supplementary Table 8**). Nevertheless, the difference in prediction ability between traits differed only marginally. Hence, we focused on the prediction abilities of the RKHS model (**Figure 6**). For predicting the facultative ICARDA accessions, the average prediction abilities of the combined spring and winter IPK populations (WS-F) exceeded those of the winter (W-F) and spring (S-F) scenarios.

To evaluate the usefulness of the across-genebank predictions, we compared the prediction abilities of the most accurate performing model found in the integrated across-genebank prediction scenario (RKHS) with those within ICARDA genebank prediction used as a benchmark scenario (GBLUP). With few exceptions (e.g., the spring-to-spring scenario of PH), prediction abilities were higher using the integrated across-genebank training dataset than within the ICARDA genebank (**Table 2**). Interestingly, two-rowed populations showed in general a greater increase in prediction abilities (131% on average) than the six-rowed populations (9.73%) by shifting from the within-genebank prediction scenario to the integrated across-genebank prediction scenario, with the most notorious case for PH of winter accessions (almost eightfold improvement).

**Table 2:** Percentage change (%) in the prediction abilities of the best-performing models in the integrated across-genebank predictions over and within ICARDA genebank predictions for heading date (HD; days), plant height (PH; cm), and thousand kernel weight (TKW; g) across the different growth habits.

Trait	scenario 3 (RKHS) vs scenario 1 (GBLUP)	r-all	2-rowed	6-rowed
**HD**	W_W vs winter	**↑** 8.87	**↑** 38.60	**↑** 2.50
	S_S vs spring	**↑** 11.56	**↑** 41.13	**↑** 13.55
	WS_F vs facultative	**↑** 0.25	**↓** -6.90	**↑** 2.51
**PH**	W_W vs winter	**↑** 50.11	**↑** 888.42	**↑** 31.15
	S_S vs spring	**↓** -20.10	**↓** -0.09	**↓** -18.26
	WS_F vs facultative	**↑** 34.00	**↑** 149.57	**↑** 27.25
**TKW**	W_W vs winter	**↑** 3.73	**↑** 32.30	**↑** 12.64
	S_S vs spring	**↑** 7.19	**↑** 40.26	**↑** 7.49
	WS_F vs facultative	**↑** 7.23	**↓** -3.92	**↑** 8.70

The results were expressed in terms of the percent of increase or decrease for each respective growth-type and row-type subpopulations across the traits.

RKHS, reproducing kernel Hilbert space regression; GBLUP, genomic best linear unbiased prediction; W_W, winter to winter; S_S, spring to spring; WS_F, winter and spring to facultative.

## Discussion

Genebanks are considered a reservoir of untapped genetic diversity for potential climate-relevant traits and improved adaptation to various biotic and abiotic stresses (Anglin et al., 2018; Guerra et al., 2022; Leigh et al., 2022). Phenotypic characterizations and documentation of genebank material are essential to promote the effective use of plant genetic resources because without them, searching for valuable accessions with desirable agronomic traits is like searching blindfolded for the proverbial needle in a haystack (Mascher et al., 2019). However, the genetic landscape that genebank managers must navigate to access information of their accessions is labor- and resource-intensive. As an interesting alternative, we explored the potential of genome-wide predictions to overcome the phenotyping bottleneck and hence unlock the genetic merits of plant genetic resources in two genebanks. Comprehensive historical data from IPK and ICARDA on flowering/heading date, plant height, and thousand kernel weight collected during seed regeneration cycles were used to demonstrate the combined powers of across-genebank predictions to support genebanks with trait information on accessions.

### Genome-wide prediction is a powerful tool to fill gaps in genebank information

Genome-wide predictions for ICARDA accessions were conducted at two levels: predictions within and across genebanks. Within the ICARDA genebank (**Figure 3**), which was set as a benchmark scenario, the prediction ability of heading date, plant height, and thousand kernel weight is positively associated with their heritability (**Table 1**). This relationship between prediction ability and heritability has been reported previously (de Oliveira et al., 2018; Arojju et al., 2020). Moreover, despite the statistical model applied, prediction abilities differed only marginally with GBLUP showing a slight advantage over other models (**Supplementary Table 6**). Therefore, we can propose the use of GBLUP as the default genomic prediction model to impute phenotypic values within a genebank. For the one-sided across-genebank predictions, despite the large differences in prediction ability between traits, the difference in prediction abilities was less pronounced between models (**Supplementary Table 7**). However, the best-performing model (EGBLUP), accounting for additive-by-additive epistasis, did not show sufficient prediction performance within the ICARDA genebank (**Supplementary Figure 1**). Interestingly, the integrated approach clearly underlined the contribution of the borrowed information from the ICARDA genebank to enhance the prediction ability (**Table 2**), hence making it more promising for predictions across two contrasting genebanks. Therefore, genome-wide prediction can be an excellent alternative to populate genebanks with phenotypic estimates in a cost- and time-effective way. This will help to bridge gaps between genebanks, enrich genebank information, and help in capturing the genetic diversity and allelic richness present across genebank collections. In the same context, we have demonstrated the profit of genome-wide prediction to predict the facultative type across genebanks using the pooled spring and winter populations and, hence, unlock the valuable diversity of this unique growth habit that provides the flexibility to be sown either in the fall as winter or even as a spring crop. Considering the relatively limited number of facultative types in the IPK collection, a promising approach would be to predict the growth habit of accessions with missing information, effectively extending the population size and, hence, bolstering predictive abilities. Furthermore, an intriguing alternative to genome-wide prediction would be the utilization of functional markers for classification. However, the current use of GBS data poses limitations, preventing a detailed functional marker-based classification, particularly for the haplotypes at VRN-H2 and VRN-H1. Nonetheless, we are optimistic that this limitation can be addressed with an increased density of genomic information. By leveraging genotypic data that incorporates information from functional markers with genome-wide prediction abilities, we could develop a compelling strategy that holds great potential for precise growth habit predictions. With these innovative methods on the horizon, we anticipate gaining a deeper and more nuanced understanding of growth habits in genebank accessions. The continuous advancement of genomic technologies and functional marker applications will undoubtedly pave the way for a new era of precision in predicting growth habits, fostering significant progress in barley breeding and crop management strategies.

### Pervasive interaction between genotypes and target environments impacts across-genebank prediction

On a large scale, we observed that the IPK genebank covers most of the neutral molecular diversity existent among the portion genotyped of the ICARDA collection (**Figure 2**). In addition, for most evaluated traits across the different growth habits, modeling a population-structure-related covariable like row type did not improve predictabilities for ICARDA accessions (**Figure 4**). This suggests that other factors beyond population structure are influencing predictions for the ICARDA genebank and limit the prediction ability for situations where phenotypic data are exclusively available in one genebank. To gain more knowledge about this and minimize any confounding effect of population structure, we further explored the phenotypes of ICARDA-like IPK accessions and their close relatives from the ICARDA genebank (Euclidean distance< 0.01; **Supplementary Figure 2**). In the best case, the imperfect correlations between relative pairs were 0.63, 0.41, and 0.68 for HD, PH, and TKW, respectively. In fact, ICARDA accessions were phenotyped in the Central West Asia North Africa (CWANA) region, where the environmental conditions differ significantly from the European weather conditions due to frequent drought and terminal heat stress. We thus conclude that phenotypic plasticity as a result of the interaction between genotypes and the environment could be one of the main factors reducing the connectivity between training and test sets in across-genebank prediction scenarios.

### Trait heritability and environment connectivity: two essential factors to improve prediction ability across genebanks

Genetic resources are vital for future food security. The deployment of advanced technologies would provide an unprecedented opportunity to profit from the immense natural diversity stored in genebanks. Following the successful proof-of-concept implementation of genome-wide predictions within genebank accessions (Crossa et al., 2016; Yu et al., 2016; Kehel et al., 2020; Jiang et al., 2021; Schulthess et al., 2022), we expanded this integrative strategy to a broader context for the enrichment of genebank phenotypic information across genebanks. The integrated across-genebank prediction was successfully applied to estimate the breeding value across two contrasting genebanks using a larger population size and larger marker density. However, the results were conditioned by two main factors: trait heritability and connectivity of the training population to the test set. To capture the variance resulting from the genotype × environment interaction (GEI), methods have been developed and applied to fit detailed variables in the models and deal with genetic/environmental heterogeneity within datasets (Crossa et al., 2022; Rogers and Holland, 2022). From our observations, we assume that a proportion of accessions from the ICARDA genebank with similar environmental features was informative enough to improve the prediction abilities. Particularly for historical multi-locations data, it would be also worthwhile to deploy models that take into consideration the time-series/spatial structure of different environmental conditions. Alternatively, due to the large effect of weather variables on the genotypic response of accessions held globally in genebanks, grouping the accessions into a small number of clusters with similar features (e.g., climate patterns and trial management) might be a feasible approach to identify mega-environments driving their separation. The identification of these mega-environments could provide useful information for optimized training populations and thus improve the prediction accuracy across genebanks.

## Data availability statement

All relevant phenotypic data presented in the study are included in the **Supplementary Material**. The associated genotypic data and a sample R code of the three prediction models used to carry out the one-sided across-genebank prediction using a sample data can be accessed via the e!DAL online repository (http://dx.doi.org/10.5447/ipk/2023/8).

## Author contributions

YJ and JR designed the study and edited the manuscript. SE and YJ performed the data analysis. SE wrote the manuscript. ZK and AA provided the material and reviewed the manuscript. AS, YZ, MM, and NS reviewed and edited the manuscript. MM, NS, and AH generated and possessed genomic data. MH prepared variant matrices. All authors contributed to the article and approved the submitted version.
