# Supplementary figures and images for: Genomic predictions to leverage phenotypic data across genebanks
Source: Front Plant Sci. 2023 Aug 28;14:1227656. doi: 10.3389/fpls.2023.1227656 (PMC10493331; doi:10.3389/fpls.2023.1227656)

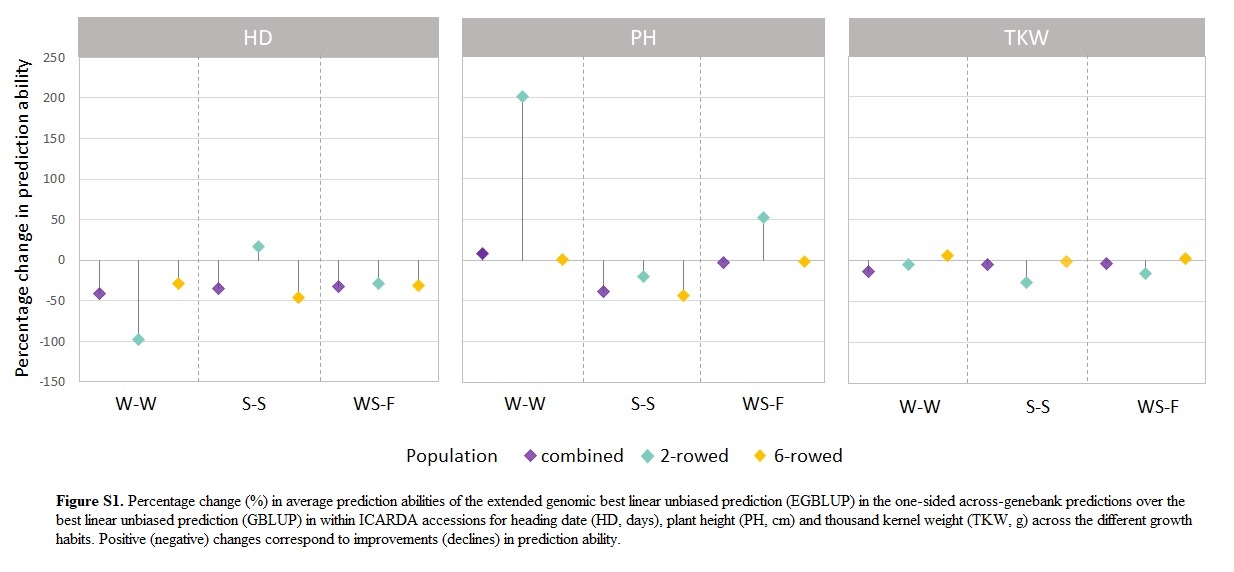

Supplement: Supplementary file 1 [file Image_1.jpg]

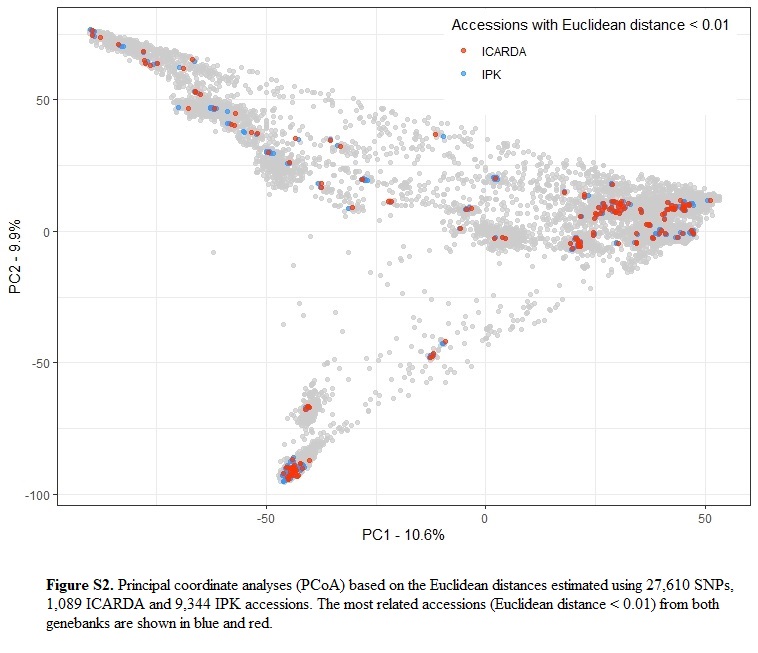

Supplement: Supplementary file 2 [file Image_2.jpeg]
